# A Limonoid, 7-Deacetoxy-7-Oxogedunin (CG-1) from Andiroba (*Carapa guianensis*, Meliaceae) Lowers the Accumulation of Intracellular Lipids in Adipocytes via Suppression of IRS-1/Akt-Mediated Glucose Uptake and a Decrease in GLUT4 Expression

**DOI:** 10.3390/molecules24091668

**Published:** 2019-04-28

**Authors:** Chihiro Matsumoto, Atsushi Koike, Reiko Tanaka, Ko Fujimori

**Affiliations:** 1 Department of Pathobiochemistry, Osaka University of Pharmaceutical Sciences, 4-20-1 Nasahara, Takatsuki, Osaka 569-1094, Japan; e17904@gap.oups.ac.jp (C.M.); koike@gly.oups.ac.jp (A.K.); 2 Department of Medicinal Chemistry, Osaka University of Pharmaceutical Sciences, 4-20-1 Nasahara, Takatsuki, Osaka 569-1094, Japan; tanakar@gly.oups.ac.jp

**Keywords:** adipocyte, andiroba, GLUT4, IRS-1/Akt, limonoid

## Abstract

Limonoids are phytochemicals with a variety of biological properties. In the present study, we elucidated the molecular mechanism of suppression of adipogenesis in adipocytes by a limonoid, 7-deacetoxy-7-oxogedunin (CG-1) from *Carapa guianensis* (Meliaceae), known as andiroba. CG-1 reduced the accumulation of intracellular triglycerides in a concentration-dependent manner. The expression levels of the adipogenic, lipogenic, and lipolytic genes were decreased by CG-1 treatment, whereas the glycerol release level was not affected. When CG-1 was added into the medium during days 0-2 of 6-days-adipogenesis, the accumulation of intracellular lipids and the mRNA levels of the adipogenesis-related genes were decreased. In addition, the phosphorylation level of insulin receptor substrate-1 (IRS-1) and Akt in the early phase of adipocyte differentiation (within 1 day after initiating adipocyte differentiation) was reduced by CG-1. Furthermore, insulin-activated translocation of glucose transporter 4 to the plasma membranes in adipocytes was suppressed by CG-1, followed by decreased glucose uptake into the cells. These results indicate that an andiroba limonoid CG-1 suppressed the accumulation of intracellular lipids in the early phase of adipocyte differentiation through repression of IRS-1/Akt-mediated glucose uptake in adipocytes.

## 1. Introduction

Obesity is now a public health problem both in developed and developing countries [1]. Obesity is defined as an abnormal increase in adipose tissue mass, and is associated with the occurrence of lifestyle-related diseases such as hyperlipidemia, diabetes mellitus, hypertension, and cardiovascular disease [1,2,3]. Adipocytes are specialized cells that store lipids as an energy source to regulate lipid metabolism and energy balance in the body. However, an excess amount of lipid accumulation in adipose cells results in obesity. Therefore, elucidation of the regulation mechanism of adipocyte differentiation is critical to control adipogenesis and obesity and to develop anti-obesity medicines.

Adipocyte differentiation is influenced by various factors such as hormones, growth factors, gene expression, and environmental conditions. Adipocyte differentiation (adipogenesis) proceeds via a multi-step process, which includes a cascade of various transcription factors and cell-cycle proteins [4]. During adipogenesis, several key transcription factors such as CCAAT/enhancer-binding proteins (C/EBPs), peroxisome proliferator-activated receptor γ (PPARγ), and sterol regulatory element-binding protein-1 (SREBP-1) play central roles in the regulation of adipocyte differentiation by controlling the transcription of many types of adipogenesis-related genes [4,5].

Insulin is also important in the control of adipogenesis. Insulin regulates a variety of physiological effects such as metabolisms, cell growth, and differentiation in a variety of cells and tissues [6]. The skeletal muscle, adipose tissue, and liver are major target tissues of insulin [7]. However, the actions of insulin are different in each tissue [7]. In adipose tissue, insulin enhances the intracellular uptake of glucose [8] and fatty acids, and inhibits lipolysis [9]. Insulin binds with insulin receptors (IRs) on the cell surface, followed by promoting phosphorylation of the IR itself and insulin receptor substrates (IRSs). Subsequently, phosphorylated IRS-1 activates the downstream molecules such as phosphoinositide 3-kinase (PI3K) and Akt, known as protein kinase B. Then, Akt activates various events such as the translocation of glucose transporters (GLUT) to the plasma membranes to promote intracellular glucose uptake [6].

Limonoids are phytochemicals that are abundantly present in citrus fruits [10]. Citrus limonoids are highly oxygenated triterpenoids that are mainly found in the Rutaceae and Meliaceae families [11]. Limonin was the first identified and is the most abundant limonoid in citrus [12]. *Carapa guianensis* Aublet, known as andiroba, belongs to the family Meliaceae, and is a large neotropical tree found mainly in South America [13,14]. The oil from andiroba seeds is used as a medicinal plant in the Amazon rainforest region [15]. Andiroba seeds display a variety of biological activities; i.e., anti-malarial [16], anti-allergy [17,18], anti-inflammatory [19], and antioxidant [20] effects. Andiroba seeds are rich in limonoids [21], and various limonoids have been isolated from the seeds of andiroba. Among them, 7-deacetoxy-7-oxogedunin (CG-1) is a major limonoid in the seeds of andiroba that inhibited LPS-induced activation of macrophages and decreased sensitivity to tumor necrosis factor-α in hepatocytes [22]. In addition, some limonoids, e.g., nomilin [11], obacunone [23], ceramicine B [24], and kihadanin B [25] showed anti-adipogenic and anti-obesity effects. Thus, it can be expected that a limonoid CG-1 has also a variety of biological activities. In the present study, we investigated the anti-adipogenic effect of a limonoid CG-1 from andiroba seeds and elucidated its molecular mechanism in adipocytes.

## 2. Results

### 2.1. Extraction, Purification, and Structural Identification of the Limonoid CG-1

**Figure 1:**
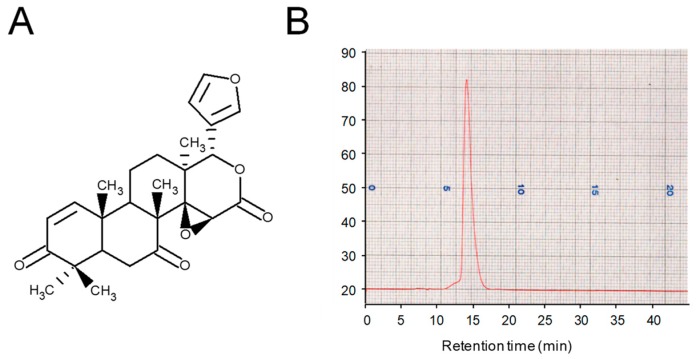
Purification of andiroba limonoid CG-1. (**A**) Chemical structure of the limonoid CG-1. (**B**) Chromatogram of CG-1 purified from the seeds of andiroba (*C. guianensis*, Meliaceae) by HPLC.

Andiroba limonoids were extracted from the seeds of *C. guianensis*, Meliaceae with methanol and purified as described previously [26]. The limonoid CG-1 (Figure 1A) was purified from andiroba seeds, and the purity was determined by HPLC with refractive index detection. The purity of isolated CG-1 was at least 99% (Figure 1B). The chemical structure was confirmed by nuclear magnetic resonance (data not shown).

### 2.2. Decrease in Lipid Accumulation by CG-1

**Figure 2:**
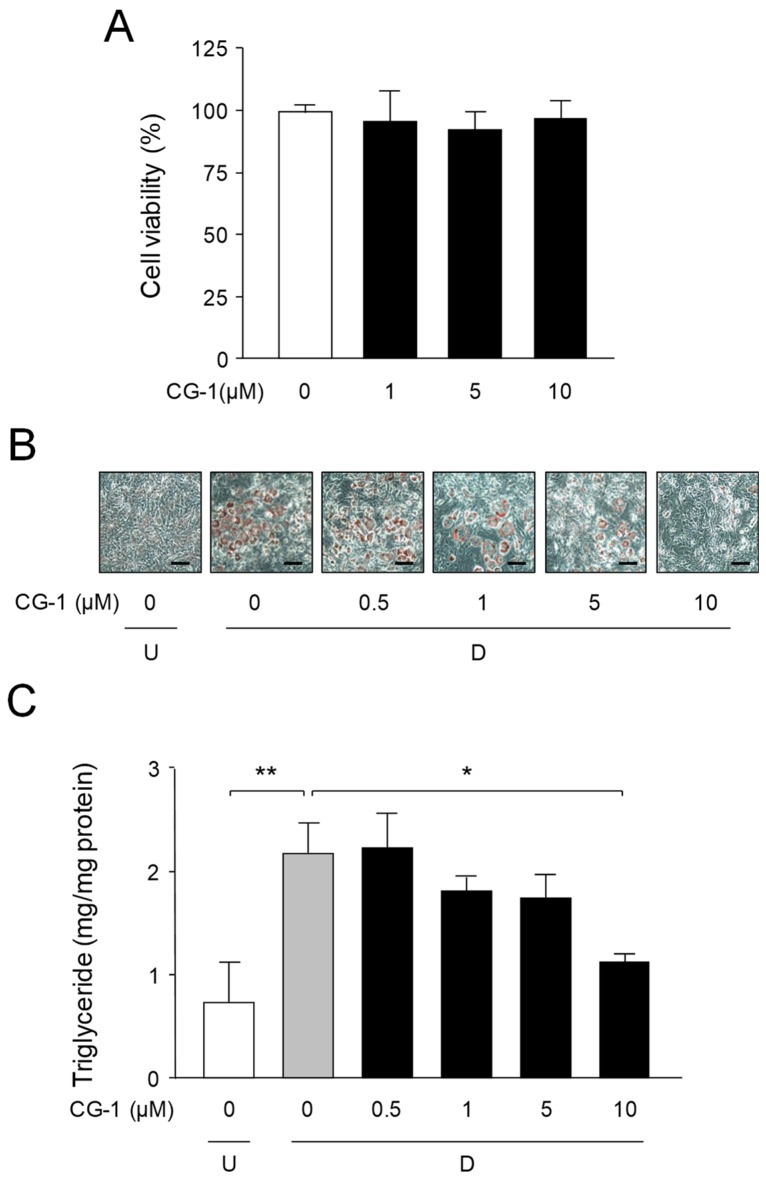
Suppression of intracellular lipid accumulation by CG-1. (**A**) Cytotoxicity of CG-1 in 3T3-L1 cells. The cells were incubated for 6 days in DMEM with various concentrations of CG-1 (0-10 μM). Data show the means ± S.D. from three experiments. (**B**) Oil Red O staining of intracellular lipid droplets. 3T3-L1 cells (undifferentiated cells: U) were differentiated into adipocytes (D) for 6 days in DMEM with various concentrations of CG-1 (0–10 μM). The cells were observed by a microscope (200x). Scale bar = 200 μm. (**C**) The intracellular triglyceride level. 3T3-L1 cells (undifferentiated cells: U; *white column*) were differentiated (**D**) into adipocytes for 6 days in DMEM without (*gray column*) or with CG-1 (0.5, 1, 5, 10 μM; *black columns*). Data are presented as the means ± S.D. from three experiments. * *p* < 0.01, ** *p* < 0.05, as indicated by the brackets.

The WST-8 assay was carried out to determine the cell toxic effect of CG-1 on mouse 3T3-L1 cells. No significant cytotoxicity was observed at concentrations up to 10 μM CG-1 (Figure 2A). Next, we investigated the effect of CG-1 on adipocyte differentiation and on the lipid accumulation in 3T3-L1 cells. The cells were differentiated into adipocytes in the medium containing various concentrations of CG-1 (0–10 μM) for 6 days. Oil Red O staining showed that CG-1 lowered the accumulation of intracellular lipids in a concentration-dependent manner (Figure 2B). 

The intracellular triglyceride level was decreased in a concentration-dependent manner and was lowered by approximately 50% at 10 μM CG-1 in the adipocyte differentiated 3T3-L1 cells (Figure 2C). These results indicate that CG-1 repressed the lipid accumulation in adipocytes. Thus, we used 10 μM CG-1 in the subsequent experiments.

### 2.3. Effect of CG-1 on Expression of Adipogenic, Lipogenic, and Lipolytic Genes in Adipocytes

**Figure 3:**
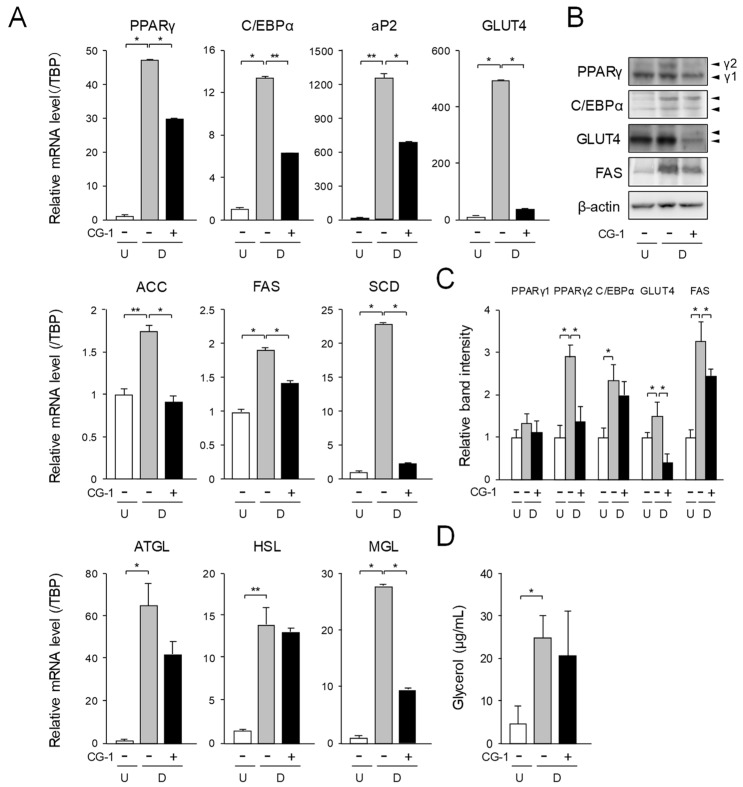
Suppression of adipogenesis by CG-1 in 3T3-L1 cells. (**A**) Transcription levels of the adipogenesis-related genes in CG-1-treated 3T3-L1 cells. The cells (undifferentiated cells: U; *white columns*) were differentiated (**D**) into adipocytes for 6 days in DMEM without (*gray columns*) or with CG-1 (10 μM; *black columns*). Results are presented as the means ± S.D. from three experiments. * *p* < 0.01, ** *p* < 0.05, as indicated by the brackets. (**B**) Change in the protein levels in 3T3-L1 cells. The cells were cultured as described in the legend of Figure 3A. Protein (15 μg) was loaded in each lane. Data are representative of three experiments. β-actin was used as the internal control. γ1 and γ2 mean PPARγ isoforms: PPARγ1 and PPARγ2, respectively. Data are representative of three experiments. (**C**) Band intensity of Western blot analysis. About C/EBPα and GLUT4, both of these two bands were measured and shown as the total in band intensity. Results are presented as the means ± S.D. * *p* < 0.01, as indicated by the brackets. (**D**) Glycerol release level in 3T3-L1 cells. The cells were differentiated as described in the legend of Figure 3A. Data are the means ± S.D. from three experiments. * *p* < 0.01, as indicated by the bracket.

To investigate the mechanisms underlying suppression of adipogenesis by CG-1 in 3T3-L1 cells, the mRNA levels of the adipogenic genes were measured by quantitative PCR. The mRNA levels of the PPARγ, C/EBPα, and their target genes such as fatty acid binding protein 4 (aP2) and GLUT4 genes were enhanced approximately 47-, 15-, 1,354-, and 512-fold, respectively, during adipogenesis (Figure 3A). 

On the contrary, when the cells were differentiated into adipocytes under the existence of CG-1 in the medium, the expression levels of these genes were decreased to about 63%, 45%, 65%, and 5%, respectively, of the vehicle-treated differentiated cells (Figure 3A). The transcription levels of the lipogenic genes such as acetyl-CoA carboxylase (ACC), FAS, and stearoyl-CoA desaturase (SCD) were enhanced by approximately 1.7-, 1.9-, and 23-fold, respectively, as compared with those of the undifferentiated cells (Figure 3A). The mRNA levels of the ACC, FAS, and SCD genes were lowered approximately 48%, 25%, and 90%, respectively, of the vehicle-treated differentiated cells by treating the cells with CG-1 (Figure 3A). In addition, the mRNA expression levels of the lipolytic genes, adipocyte triglyceride lipase (ATGL), HSL, and monoglyceride lipase (MGL) were enhanced by approximately 64-, 15-, and 27-fold, respectively, during adipogenesis (Figure 3A). In contrast, when the cells were differentiated into adipocytes in the medium with CG-1, the mRNA levels of these lipolytic genes were lowered to about 56%, 94%, and 34%, respectively, of those of the vehicle-treated differentiated cells (Figure 3A). Moreover, Western blot analysis of PPARγ, C/EBPα, GLUT4, and FAS showed almost the same results as those of the mRNA expression analysis (Figure 3B,C).

Then, we measured the glycerol release level to estimate the lipolytic ability in adipocytes. Glycerol release was increased during adipogenesis (Figure 3D). When CG-1 was added to the medium during adipogenesis of 3T3-L1 cells, the glycerol release level tended to decrease, as compared to that of the vehicle-treated differentiated cells (Figure 3D). These results, taken together, indicate that CG-1 decreased the mRNA levels of the adipogenic, lipogenic, and lipolytic genes, but did not affect lipolysis in adipocytes.

### 2.4. Suppression of Early Phase of Adipogenesis by CG-1

**Figure 4:**
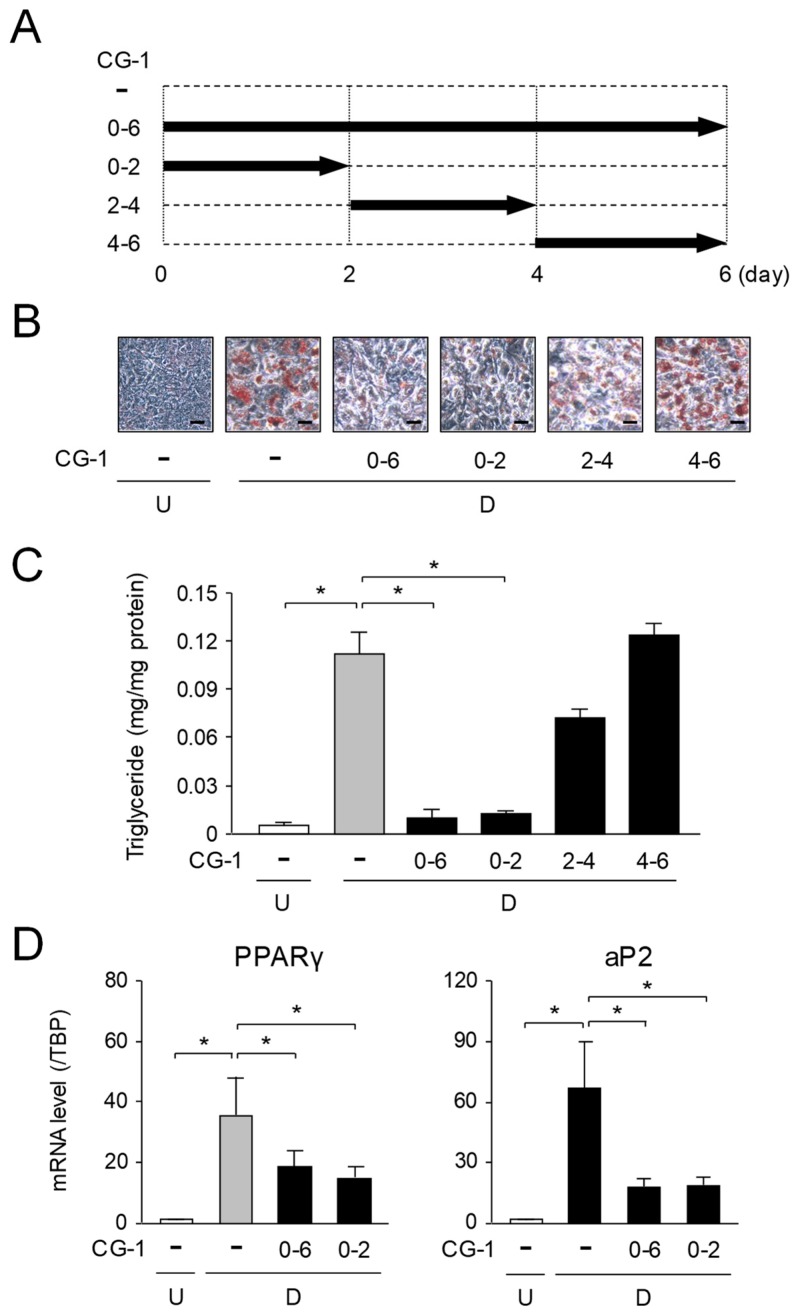
Adipocyte differentiation-phase-specific inhibition of adipogenesis by CG-1. (**A**) Addition of CG-1 into the medium at the indicated time-points in 6-days-adipogenesis. (**B**) Oil Red O staining of lipid droplets in phase-specific CG-1-treated 3T3-L1 cells. The cells (undifferentiated cells: U) were differentiated into adipocytes (differentiated cells: D) in DMEM with CG-1 (10 μM) during days 0–6, 0–2, 2–4, or 4–6 of 6-days-adipogenesis. Data are representative of three experiments. The cells were observed by a microscope (200×). Scale bar = 100 μm. (**C**) Intracellular triglyceride level in 3T3-L1 cells. The cells (undifferentiated cells: U; *white column*) were differentiated into adipocytes (differentiated cells: D) in DMEM without (*gray column*) or with CG-1 (10 μM; *black columns*) during days 0–6, 0–2, 2–4, or 4–6 of 6-days-adipogenesis. Results are shown as the means ± S.D. from three experiments. * *p* < 0.01, as indicated by the brackets. (**D**) Suppression of adipogenesis-related gene expression in the early phase of adipogenesis. Expression level of the adipogenic genes in CG-1-treated 3T3-L1 cells. The cells (undifferentiated cells: U; *white columns*) were differentiated (D) into adipocytes in DMEM without (*gray columns*) or with CG-1 (10 μM; *black columns*) during days 0–6, 0–2, 2–4, or 4–6 of 6-days-adipogenesis. Results are expressed as the means ± S.D. from three experiments. * *p* < 0.01, as indicated by the brackets.

We investigated when CG-1 suppressed adipocyte differentiation during 6-days-adipogeneisis. 3T3-L1 cells were differentiated into adipocytes in the medium containing CG-1 during days 0–2, 2–4, 4–6, or 0–6, of 6-days-adipogenesis (Figure 4A). At day 6, the intracellular lipids were stained with Oil Red O, and the intracellular triglyceride levels and the expression levels of the adipogenic genes were measured. Intracellular lipids were accumulated during adipogenesis (Figure 4B,C), and their accumulation was lowered when CG-1 was added into the medium during days 0–2 or days 0–6 of 6-days-adipogenesis (Figure 4B,C). On the contrary, when CG-1 was present in the medium during days 2–4 or days 4–6 of 6-days-adipogenesis, a significant decrease in accumulation of intracellular lipids was not observed, which was almost the same as that of the vehicle-treated differentiated cells (Figure 4B,C). Moreover, the expression levels of the adipogenic PPARγ and aP2 genes were decreased when CG-1 was added into the medium during days 0–2 and days 0–6 of 6-days-adipogenesis, whose results well resembled to those during days 0–6 of 6-days-adipogenesis (Figure 4D). While, no change in the expression of these genes was detected when CG-1 was added into the medium during days 2–4 and days 4–6 of 6-days-adipogenesis (data not shown). These results reveal that CG-1 lowered the intracellular lipid accumulation by suppressing the progression in the early phase of adipogenesis. However, the expression of C/EBPβ and C/EBPδ, which play central roles in the progression of the early phase of adipocyte differentiation was not affected by treatment with CG-1 (data not shown).

### 2.5. Inhibition of Activation of IRS-1/Akt Axis by CG-1

**Figure 5:**
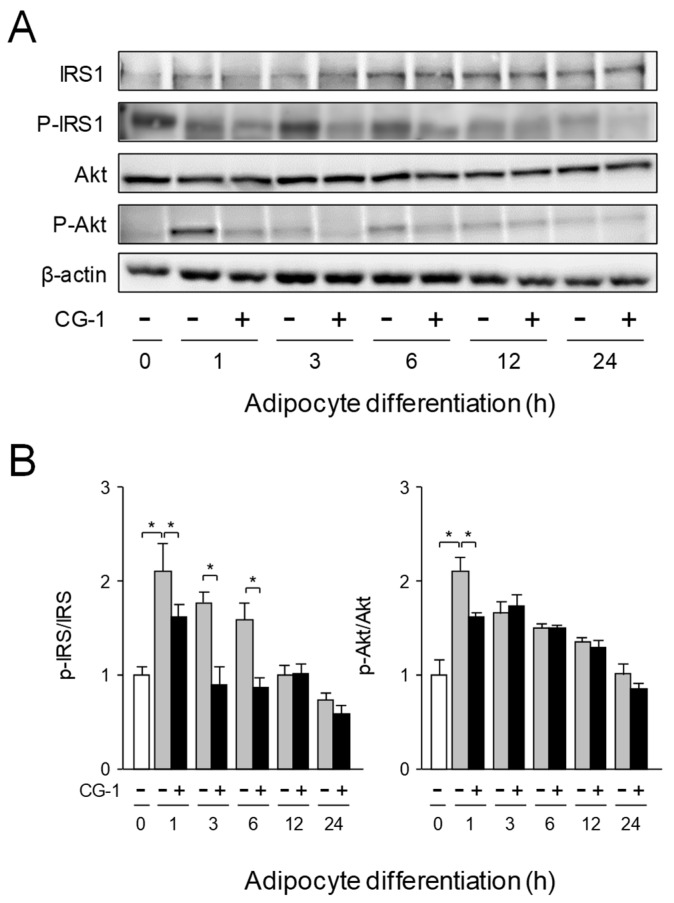
Inhibition of activation of IRS-1/Akt axis by CG-1. (**A**) Expression and phosphorylation of IRS-1, and Akt in CG-1-treated 3T3-L1 cells. The cells were differentiated into adipocytes for the indicated time in DMEM with CG-1 (0 or 10 μM). Proteins (15 μg/lane) were subjected to SDS-PAGE-Western blot analysis. Data are representative of three experiments. (**B**) Band intensity of Western blot analysis. Results are presented as the means ± S.D. * *p* < 0.01, as indicated by the brackets.

Insulin acts through the insulin receptor, which undergoes auto-phosphorylation and subsequently phosphorylates IRS-1 [27], followed by activating the PI3K/Akt pathway [28]. We investigated the effects of CG-1 on the IRS-1/Akt signaling and the suppression of adipogenesis of 3T3-L1 cells. IRS-1 was expressed in undifferentiated 3T3-L1 cells (0 h), and its expression was gradually increased up until 24 h in the presence or absence of CG-1 (Figure 5A,B). In addition, IRS-1 was phosphorylated in undifferentiated cells (0 h), and a higher phosphorylation level was maintained until 6 h after initial adipocyte differentiation, and then its phosphorylation level was gradually decreased (Figure 5A,B). In contrast, when the cells were differentiated in the presence of CG-1, phosphorylation level of IRS-1 was clearly decreased in the early phase (1-6 h after the initiation of adipocyte differentiation) of adipogenesis (Figure 5A,B). These results indicate that CG-1 suppressed the phosphorylation of IRS-1 and Akt in the insulin signaling in the early phase of adipogenesis.

### 2.6. Inhibition of Translocation of GLUT4 and Glucose Uptake by CG-1

**Figure 6:**
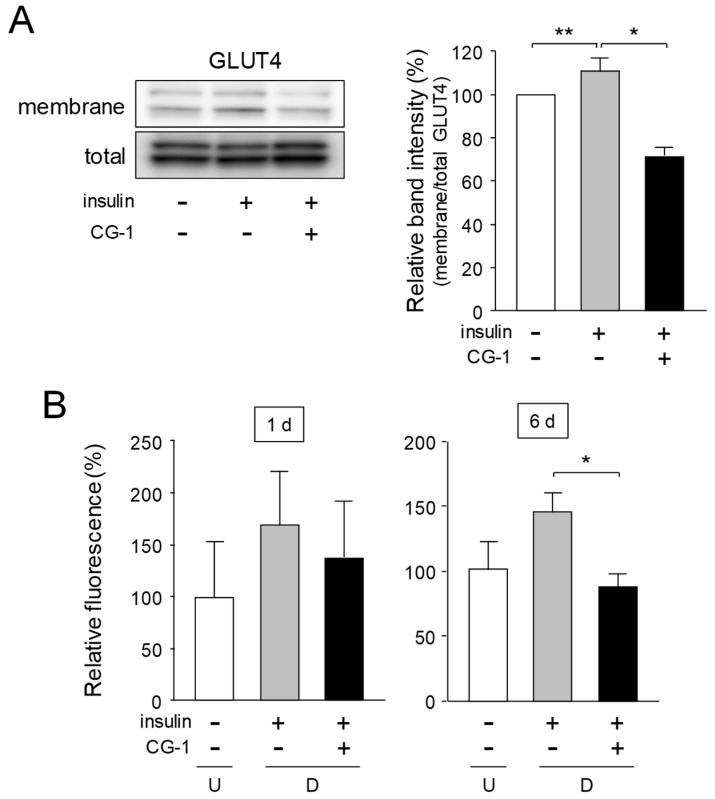
Suppression of GLUT4 translocation and glucose uptake in CG-1-treated 3T3-L1 cells. (**A**) GLUT4 protein in the membrane- and total-fractions. 3T3-L1 cells were serum starved for 16 h and cultured in DMEM with insulin (10 μg/mL) and/or CG-1 (10 μM) for 10 min after pre-treatment with CG-1 (10 μM) for 1 h. The membrane fractions (membrane) were used for Western blot analysis. Total GLUT4 (total) was detected as the input control. Protein (10 μg) was loaded in each lane. Data are representative of three experiments. Band intensity was measured and calculated by MultiGauge software. * *p* < 0.01 or ** *p* < 0.05, as indicated by the brackets. (**B**) Glucose uptake in 3T3-L1 cells. The cells (undifferentiated cells: U; *white columns*) were differentiated into adipocytes (differentiated cells: D) for 1 or 6 day in DMEM without (*gray columns*) or with CG-1 (10 μM; *black columns*). 2-NBDG uptake level was measured by a fluorescent microplate reader. Results are the means ± S.D. from three experiments. *p* < 0.01, as indicated by the bracket.

GLUT4 is generally localized in the specialized compartments in the cytosol and is translocated to the plasma membranes, in a process that is activated by insulin [29]. In order to investigate the effect of CG-1 on GLUT4 translocation in adipocytes, the level of GLUT4 translocated onto the membranes was examined by Western blot analysis. When the cells were stimulated with insulin, the GLUT4 level in the membrane fractions was notably increased as compared with that of the untreated cells (Figure 6A). In contrast, the GLUT4 level in the membranes of the insulin-treated cells was reduced when the cells were cultured in the medium containing insulin together with CG-1, although the all GLUT4 levels in total protein fractions were almost the same (Figure 6A).

Then, we measured the glucose uptake level into the cells by using a fluorescent glucose analog, 2-NBDG. The fluorescent level in the cells was enhanced by treating with insulin (Figure 6B). In contrast, when CG-1 was added to the medium prior to adding insulin, insulin-activated 2-NBDG uptake was profoundly reduced (Figure 6B). These results, taken together, reveal that CG-1 repressed the insulin-activated glucose uptake by suppressing translocation of GLUT4 onto the plasma membranes in adipocytes.

## 3. Discussion

**Figure 7:**
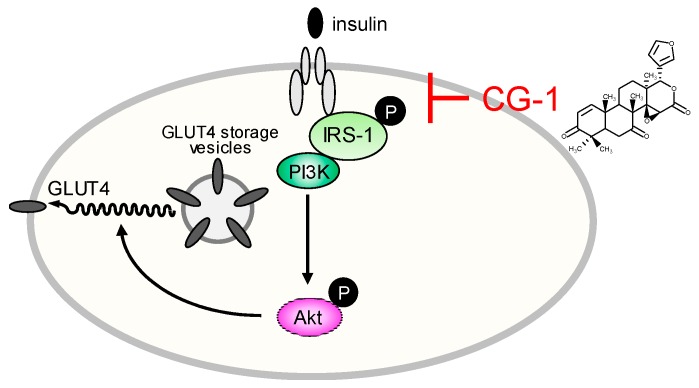
Schematic representation of CG-1-mediated suppression of intracellular lipid accumulation in 3T3-L1 adipocytes.

Andiroba (*Carapa guianensis*) belongs to the Meliaceae family of plants that grow wild throughout South America, and andiroba oil extracted from the seeds has been used in traditional medicine [14]. Andiroba oil is rich in limonoids such as 7-deacetoxy-7-oxogedunin (CG-1) [21,26]. Limonoids have a variety of physiological abilities [10] including anti-adipogenic and anti-obesity effects [11,23,24,25]. In fact, CG-1 has various physiological properties such as anti-malarial [16], anti-allergy [17,18], anti-inflammatory [19], and antioxidant [20] activities. In this study, we found that a limonoid CG-1 purified from the seeds of andiroba, *C. guianensis*, Meliaceae showed anti-adipogenic effects in mouse adipocyte 3T3-L1 cells. Our results are summarized in Figure 7.

Both transcriptional regulation and hormonal control are important in the regulation of adipogenesis. Among the critical signaling involved in the regulation of adipogenesis, insulin signaling is very important, because insulin signaling activates glucose uptake and inhibits lipolysis in adipocytes [30,31]. Activation of IRS, a family of docking molecules connecting activation of IR, is essential to activate the downstream kinase cascades, including PI3K and Akt. For activation of IRS-1, its serine residue is phosphorylated, resulting in enhanced IRS-1-associated PI3K activities [32]. Phosphorylation of IRS-1 and Akt was enhanced in the early phase of adipogenesis, but repressed by treatment with CG-1 (Figure 4A,B). Moreover, CG-1 suppressed the expression of adipogenic genes such as PPARγ and C/EBPα (Figure 3A). In addition, the expression of lipogenic enzymes was remarkably lowered by the treatment with CG-1 (Figure 6A,B). The expression of the FAS and SCD genes are regulated by PPARγ in adipocytes [33,34]. Therefore, repression of PPARγ-mediated activation of lipogenesis might be associated with the suppression of lipid accumulation by CG-1 in adipocytes. The detail mechanism of CG-1-mediated repression of lipogenesis in adipocytes should be further investigated.

Glucose uptake into the cells is regulated by GLUT proteins in mammals [35]. GLUT4 is a member of glucose transporter and highly expressed in adipose tissues and muscles. GLUT4 plays a critical role in the regulation of blood glucose homeostasis, which is stimulated by insulin [33]. In fact, adipose-specific depletion of GLUT4 causes insulin resistance [36]. Translocation of GLUT4 to the plasma membranes from intracellular GLUT4 storage vesicles in adipocytes was activated in response to insulin [33]. Insulin signaling is closely associated with a variety of diseases such as obesity, hyperglycemia, and metabolic syndrome [37,38]. Insulin is a trigger for activation of tyrosine kinase of IR, which phosphorylates the tyrosine residue of IRS [27]. Upon activation by insulin, IRS binds to several SH_2_ domain-containing proteins such as the regulatory subunit of PI3K, which subsequently activates several downstream target proteins including Akt. Translocation of GLUT4 to the plasma membranes are triggered by activated Akt. Thus, as CG-1 suppressed activation of the IRS-1/Akt axis, translocation of GLUT4 was repressed. In this study, we demonstrated that CG-1 suppressed the insulin-activated translocation of membranes of GLUT4 (Figure 6A), followed by decreasing in the glucose uptake in adipocytes (Figure 6B). In addition, C/EBPα activates the expression of GLUT4 in adipocytes [39]. CG-1 lowered the expression of C/EBPα and GLUT4 (Figure 3A). Thus, CG-1 repressed the expression of GLUT4 by down-regulation of C/EBPα. Therefore, CG-1 lowered the accumulation of intracellular lipids by suppressing insulin-activated translocation of GLUT4 through IRS-1/Akt signaling in the early phase of adipogenesis and decreasing C/EBPα-activated GLUT4 expression.

## 4. Conclusions

We demonstrate that the limonoid CG-1 from andiroba (*Carapa guianensis*, Meliaceae) seeds decreased the intracellular lipid accumulation by reducing the IRS-1/Akt-mediated glucose uptake, C/EBPα-mediated GLUT4 expression level, and by suppressing PPARγ expression in adipocytes. Thus, the IRS-1/Akt signaling-mediated glucose uptake in the early phase of adipogenesis is critical in the promotion of adipogenesis. Moreover, CG-1 suppressed PPARγ-mediated activation of lipogenesis in adipocytes. In a future study, we should aim to elucidate the in vivo function of CG-1 as an anti-obesity agent.

## 5. Materials and Methods

### 5.1. Materials

Insulin, 3-isobutyl-1-methylxanthine (IBMX), dexamethasone (Dex), and Oil Red O were obtained from Sigma (St. Louis, MO, USA). 2-Deoxy-2-[(7-nitro-2,1,3-benzoxadiazol-4-yl)amino]-D-glucose] (2-NBDG) was purchased from Cayman Chemical (Ann Arbor, MI, USA). Anti-Akt (#9272; 1:500), anti-phospho-Akt (p-Akt; Thr308; #9275; 1:500), and anti-C/EBPα (#2295; 1:1000) polyclonal antibodies were from Cell Signaling (Danvers, MA, USA). Anti-GLUT4 (G4048; 1:1000) polyclonal and β-actin (AC-15; A1978; 1:3000) monoclonal antibodies were from Sigma. Anti-PPARγ (H-100; 1:1000), anti-fatty acid synthase (FAS; H-300; 1:1000), anti-IRS-1 (C-20; 1:500), and anti-phospho-IRS-1 (Tyr-632; 1:500) polyclonal antibodies and anti-mouse, anti-rabbit, and anti-goat IgG, horseradish peroxidase-linked secondary antibodies (1:1000) were from Santa Cruz Biotechnology Inc. (Dallas, TX, USA).

### 5.2. Purification of A Limonoid CG-1 from Andiroba

CG-1 was purified from the seeds of andiroba *C. guianensis*, Meliaceae and its chemical structure was determined by nuclear magnetic resonance, as described previously [26]. The purity of CG-1 was confirmed by HPLC [JASCO, Tokyo, Japan; solvent; acetonitrile: H_2_O=60: 40, column: COSMOSIL 5C18-MS (Nacalai Tesque, Kyoto, Japan)]. The purity of CG-1 was calculated by a JASCO 807-IT integrator (JASCO).

### 5.3. Cell Culture

Mouse adipocyte 3T3-L1 cells (JCRB Cell Bank, National Institutes of Biomedical Innovation, Health and Nutrition, Osaka, Japan) were cultured at 37 °C in a humidified atmosphere containing 5% CO_2_. 3T3-L1 cells were cultured in Dulbecco’s modified Eagle’s medium (DMEM; Sigma) including 10% (*v*/*v*) fetal calf serum (FCS), and penicillin (10,000 U/mL) and streptomycin (10,000 μg/mL; Nacalai Tesque). For adipocyte differentiation, after the cells reached confluence, the cells were cultured in DMEM containing 10 μg/mL insulin, 0.5 mM IBMX, and 1 μM Dex. After another 2 days, the cells were differentiated into adipocytes in fresh DMEM containing insulin (10 μg/mL) alone for more 4 days. Medium was changed every other day. CG-1 (0–10 μM) was added into the medium when the medium was changed, unless otherwise noted. When intracellular lipid droplets were stained with Oil Red O, cells were washed with PBS, and fixed with 10% (*v*/*v*) formaldehyde in PBS for 10 min. The lipid droplets were then stained with Oil Red O solution in 60% (*v*/*v*) isopropanol at 20 °C. The subsequent observation of stained lipid droplets were done with a CKX41 microscope (Olympus, Tokyo, Japan).

### 5.4. Cytotoxicity Assay

For investigating cytotoxicity, 3T3-L1 cells were grown in a 96-well plate in DMEM with various concentrations (0–50 μM) of CG-1 for 6 days. The medium was changed every other day, and CG-1 was added into the medium when the medium was replaced. Cytotoxicity was determined colorimetrically by using a Cell Counting Kit-8 (Dojindo, Kumamoto, Japan) according to the methods subscribed by the supplier.

### 5.5. Intracellular Triglyceride Level

3T3-L1 cells were differentiated into adipocytes for 6 days in DMEM in the presence or absence of CG-1. Intracellular triglyceride levels were measured by using a WAKO LabAssay Triglyceride Kit (FUJIFILM Wako Pure Chemical, Osaka, Japan). Protein concentrations were determined by the use of a Pierce BCA Protein Assay Reagent (Thermo Fisher Scientific, Waltham, MA, USA). The intracellular triglyceride concentration was normalized to the protein concentration.

### 5.6. Quantitative PCR

Total RNA was extracted by using RNAiso Plus (Takara-Bio, Kyoto, Japan) according to the manufacturer’s instructions. The quality and concentration of total RNA were determined spectrophotometrically by using a NanoDrop Lite UV-Vis Spectrophotometer (Thermo Fisher Scientific). Total RNA (1 μg) was reverse-transcribed to cDNAs by using ReverTra Ace reverse transcriptase (Toyobo, Osaka, Japan) and random hexamer primers (Takara-Bio) according to the manufacturer’s protocols. Quantitative PCR was performed in an Applied Biosystems 7500 Real Time PCR System (Thermo Fisher Scientific) using Power SYBR Green Master Mix (Thermo Fisher Scientific) and the primer sets (Table 1). The comparative Ct method was employed to calculate the relative mRNA level of the desired gene [40], and values were normalized to that of the TATA-binding protein (TBP) gene.

### 5.7. Glycerol Release Assay

3T3-L1 cells were differentiated into adipocytes for 5 days in DMEM in the presence or absence of CG-1 (10 μM). At day 5, the medium was changed to phenol red-free DMEM (Sigma) including insulin together with or without CG-1 (10 μM), and the cells were further cultured for one day. On day 6, the medium was collected and the levels of glycerol were measured by using a Free Glycerol Assay Reagent (Cayman Chemical) according to the protocols prescribed by the supplier.

### 5.8. Western Blot Analysis

The cell lysates were prepared as described [41]. Protein concentrations were calculated as described above. Proteins were subjected to SDS-PAGE gels, and transferred to polyvinylidene difluoride membranes (Immobilon; Merck Millipore, Billerica, MA, USA). The blots were blocked for 1 h in Blocking One (Nacalai Tesque), and incubated with appropriate primary antibody in Tris-buffered saline containing 0.1% (*v*/*v*) Tween 20 for 1 h, followed by incubated with secondary antibody, horseradish peroxidase-conjugate for 1 h. Immunoreactive signals were detected by using ImmunoStar LD (FUJIFILM Wako Pure Chemical) or ECL Prime Western Blotting Detection Reagent (GE Healthcare, Buckinghamshire, UK) and an LAS-3000 Luminoimage Analyzer (FUJIFILM, Tokyo, Japan). Densitometric analysis of the bands was performed by using Multi Gauge software (FUJIFILM) and Image J software [42].

### 5.9. GLUT4 Translocation Assay

Preparation of membrane fractions for the detection of GLUT4 was performed as described previously [43]. Briefly. 3T3-L1 cells were serum starved for 16 h, and prior to addition of 10 μg/mL insulin into the medium, the cells were pre-treated with CG-1 (10 μM) for 1 h. Cells were incubated in DMEM with or without 10 μg/mL insulin and/or 10 μM CG-1 for 10 min. Cells were washed twice with PBS(-), collected, and homogenized in lysis buffer consisting of 50 mM Tris-Cl (pH 8.0), 0.5 mM DTT, 0.1% (*v*/*v*) Nonidet-P40, and protease inhibitor cocktails (total protein fractions). After centrifugation (1000× *g*, 4 °C for 10 min), the pellets were dissolved in Tris buffer (50 mM, pH 8.0) containing 0.5 mM DTT and protease inhibitor cocktails, followed by centrifugation again (1000× *g*, 4 °C for 10 min). The pellets were re-dissolved in buffer containing 50 mM Tris-Cl (pH 8.0), 0.5 mM DTT, 1% (*v*/*v*) Nonidet-P40, and protease inhibitor cocktails, and then centrifugation (16,000× *g*, at 4 °C for 20 min) was carried out. The resultant supernatant fractions were further used as the membrane fractions. Measurement of protein concentrations and Western blot analysis were performed as described above.

### 5.10. Glucose Uptake Assay

To investigate whether CG-1 affects glucose uptake into adipose cells, we performed a glucose uptake assay by using 2-NBDG, a fluorescent glucose analog. 3T3-L1 cells were differentiated into adipocytes in DMEM (high glucose: 4500 mg/L; Sigma) containing 10% (*v*/*v*) FCS and antibiotics as described above. At day 6, the medium was changed to DMEM (low glucose: 1000 mg/L; Sigma) containing antibiotics alone for 20 min. After discarding the medium, the cells were washed twice with Hank’s Balanced Salt Solution (HBBS)(+) and incubated in HBSS(+) containing insulin (10 μg/mL) for 20 min, followed by adding 2-NBDG (150 μg/mL) and incubating the cells for more 30 min. The fluorescence level was measured using a multimode plate reader (Enspire 2300; excitation and emission wavelengths of 485 nm and 535 nm, respectively; PerkinElmer, Waltham, MA, USA).

### 5.11. Statistical Analysis

Data are presented as the means ± standard deviation (S.D.). Comparisons of multiple groups were analyzed by one-way ANOVA and a Tukey’s post-hoc test. To determine significant differences between 2 groups, comparisons were made using Student’s *t* tests. *p* < 0.05 was considered significant.

## Figures and Tables

**Table 1:** Nucleotide sequences of primers used in quantitative PCR.

Gene	Accession No. *	Forward Primer	Reverse Primer
PPARγ	NM_011146	5’-CAAGAATACCAAAGTGCGATCAA-3′	5′-GAGCTGGGTCTTTTCAGAATAATAAG-3′
C/EBPα	NM_007678	5′-CTGGAAAGAAGGCCACCTC-3′	5′-AAGAGAAGGAAGCGGTCCA-3′
aP2	NM_024406	5′-CAGCCTTTCTCACCTGGAAG-3′	5′-TTGTGGCAAAGCCCACTC-3′
GLUT4	NM_009204	5′-GACGGACACTCCATCTGTTG-3′	5′-GCCACGATGGAGACATAGC-3′
ACC	NM_133360	5′-GCGTCGGGTAGATCCAGTT-3′	5′-CTCAGTGGGGCTTAGCTCTG-3′
FAS	NM_007988	5′-GTTGGGGGTGTCTTCAACC-3′	5′-GAAGAGCTCTGGGGTCTGG-3′
SCD	NM_009127	5′-CGTCTGGAGGAACATCATTCT-3′	5′-CAGAGCGCTGGTCATGTAGT-3′
ATGL	NM_001163689	5′-TGACCATCTGCCTTCCAGA-3′	5′-TGTAGGTGGCGCAAGACA-3′
HSL	NM_010719	5′-GCACTGTGACCTGCTTGGT-3′	5′-CTGGCACCCTCACTCCATA-3′
MGL	NM_011844	5′-TCGGAACAAGTCGGAGGT-3′	5′-TCAGCAGCTGTATGCCAAAG-3′
TBP	NM_013684	5′-GTGATGTGAAGTTCCCCATAAGG-3′	5′-CTACTGAACTGCTGGTGGGTCA-3′

* DDBJ/ENA/GenBank database.
